# Bufalin reverses ABCB1-mediated drug resistance in colorectal cancer

**DOI:** 10.18632/oncotarget.18225

**Published:** 2017-05-26

**Authors:** Ze-Ting Yuan, Xiao-Jing Shi, Yu-Xia Yuan, Yan-Yan Qiu, Yu Zou, Cheng Liu, Hui Yu, Xue He, Ke Xu, Pei-Hao Yin

**Affiliations:** ^1^ Interventional Cancer Institute of Chinese Integrative Medicine, Putuo Hospital, Shanghai University of Traditional Chinese Medicine, Shanghai 200062, China; ^2^ Department of General Surgery, Putuo Hospital, Shanghai University of Traditional Chinese Medicine, Shanghai 200062, China

**Keywords:** bufalin, colorectal cancer, multidrug resistance, ABC transporters, ABCB1

## Abstract

Multidrug resistance (MDR), mainly mediated by ABCB1 transporter, is a major cause for chemotherapy failure. Bufalin (BU), an active component of the traditional Chinese medicine chan’su, has been reported to have antitumor effects on various types of cancer cells. The purpose of this present study was to investigate the reversal effect of BU on ABCB1-mediated multidrug resistance in colorectal cancer. BU at safe concentration (5, 10, 20 nM) could reverse chemosensitivity of ABCB1-overexpression HCT8/ADR, LoVo/ADR and HCT8/ABCB1 nearly back to their parental cells level. In addition, results from the drug accumulation studies revealed that BU was able to enhance intracellular accumulation of doxorubicin (DOX) and Rhodamine 123 (Rho-123) in a dose-dependent manner. Furthermore, Western blot assays showed that BU significantly inhibited the expression level of ABCB1 protein. Meanwhile, BU stimulated the ATPase activity of ABCB1, which suggested that BU might be a substrate of ABCB1. More interestingly, docking analysis predicted that BU could be docked into the large hydrophobic drug-binding cavity of human ABCB1. Importantly, BU remarkable increased the effect of DOX against the ABCB1 resistant HCT8/ADR colorectal cell xenografts in nude mice, without inducing any obvious toxicity. Overall, we concluded that BU efficiently reversed ABCB1-mediated MDR through not only inhibited the efflux function of ABCB1, but also down-regulate its protein expression, which might represent a potential and superior ABCB1 modulator in colorectal cancer.

## INTRODUCTION

Multidrug resistance (MDR) is the main reason for the failure of cancer chemotherapy[1]. MDR refers to the concurrent development of cross-resistance to structurally and mechanistically different anticancer drugs by tumour cells[2]. The mechanisms of MDR are complex, including overexpression of ATP-binding cassette (ABC) transporter, apoptosis inducing, autophagy inducing, DNA damage and repair, and epigenetic regulation and so on[3]. The export of chemotherapy drugs from cancer cells by adenosine triphosphate (ATP)-binding cassette (ABC) transporters is the most important mechanism of MDR[4]. The ABC transporters mainly include ABCB1 (P-glycoprotein/P-gp), ABCC1 (Multidrug resistance-associated protein/MRP1) and ABCG2 (Breast cancer resistance protein/BCRP)[5]. Among these transporters, ABCB1 is the most important resistance-inducing protein[6]. It is located on the cell membrane and is an energy-dependent drug efflux pump. ABCB1 binds to chemotherapy drugs that enter tumour cells and expels them from the cell. This action significantly lowers the drug concentration below the effective dose, thereby leading to tumour cell resistance[7, 8]. The substrates of ABCB1 include chemotherapy drugs such as vinca alkaloids, taxanes, epipodophyllotoxins and so on[8].

MDR can be reversed by developing anticancer drugs that can avoid or antagonise the tumor resistance mechanisms. This can also be achieved by combining anticancer drugs with certain types of tumour MDR reversal agents to antagonise or eliminate tumour resistance[9]. MDR reversal agents, also known as MDR regulators or chemotherapy sensitizers, are a class of non-toxic or relatively low toxic substances. At concentrations that do not affect the inhibition of tumour cell growth, MDR reversal agents act by inhibiting the expression of MDR-related genes or the function of related proteins and, thereby, completely or partially restore the sensitivity of cells to anticancer drugs[10]. Nowadays, ABCB1 inhibitors have been classified into three generations[11]. The first-generation ABCB1 inhibitors include verapamil, cyclosporine A, quinine and quinidine[12]. Examples of second- generation ABCB1 inhibitors include valspodar (PSC 833) and biricodar (VX-710)[13]. The typical third-generation ABCB1 inhibitors are tariquidar (XR9576) and zosuquidar (cyclopropyldibenzosuberane, LY335979)[14, 15]. Although a few compounds are already undergoing clinical trials, there are currently no officially approved MDR reversal agents for clinical use.

Chinese medicine and its monomers have thousands of years of history for anticancer therapy. The unique advantages of Chinese medicines have encouraged screening to identify effective and low-toxicity monomer compounds for MDR reversal and prevention, and this has become a hot topic in chemotherapy research[16, 17]. Bufalin (BU), which is the main active ingredient in Chinese toad venom, induces apoptosis of numerous types of tumour cells including those of colon, stomach, liver, and pancreas[18, 19]. In recent years, research has shown that BU can overcome MDR through multiple pathways, including the activation of apoptotic pathways and down-regulation of multidrug resistance-associated protein 1 (MRP1) expression[20–22]. However, there are currently no studies reporting the role of BU in ABCB1-mediated MDR. Our preliminary *in vitro* screening experiments revealed that BU played a role in reversing tumour MDR. The results of docking experiments showed that BU and ABCB1 transporter had good interactions, and we speculated that it might have the potential to reverse MDR. Therefore, the aim of this study was to further elucidate the activity and mechanism of action of BU in the reversal of ABCB1-mediated MDR. Drug-sensitive and -resistant colon cancer cell lines and colon cancer xenografts of nude mice were selected as the *in vitro* and *in vivo* models, respectively. This study is the first to report the selective reversal of ABCB1-mediated MDR by BU in colon cancer.

## RESULTS

### BU sensitized ABCB1-overexpressing cells to chemotherapeutic drugs

**Figure 1:**
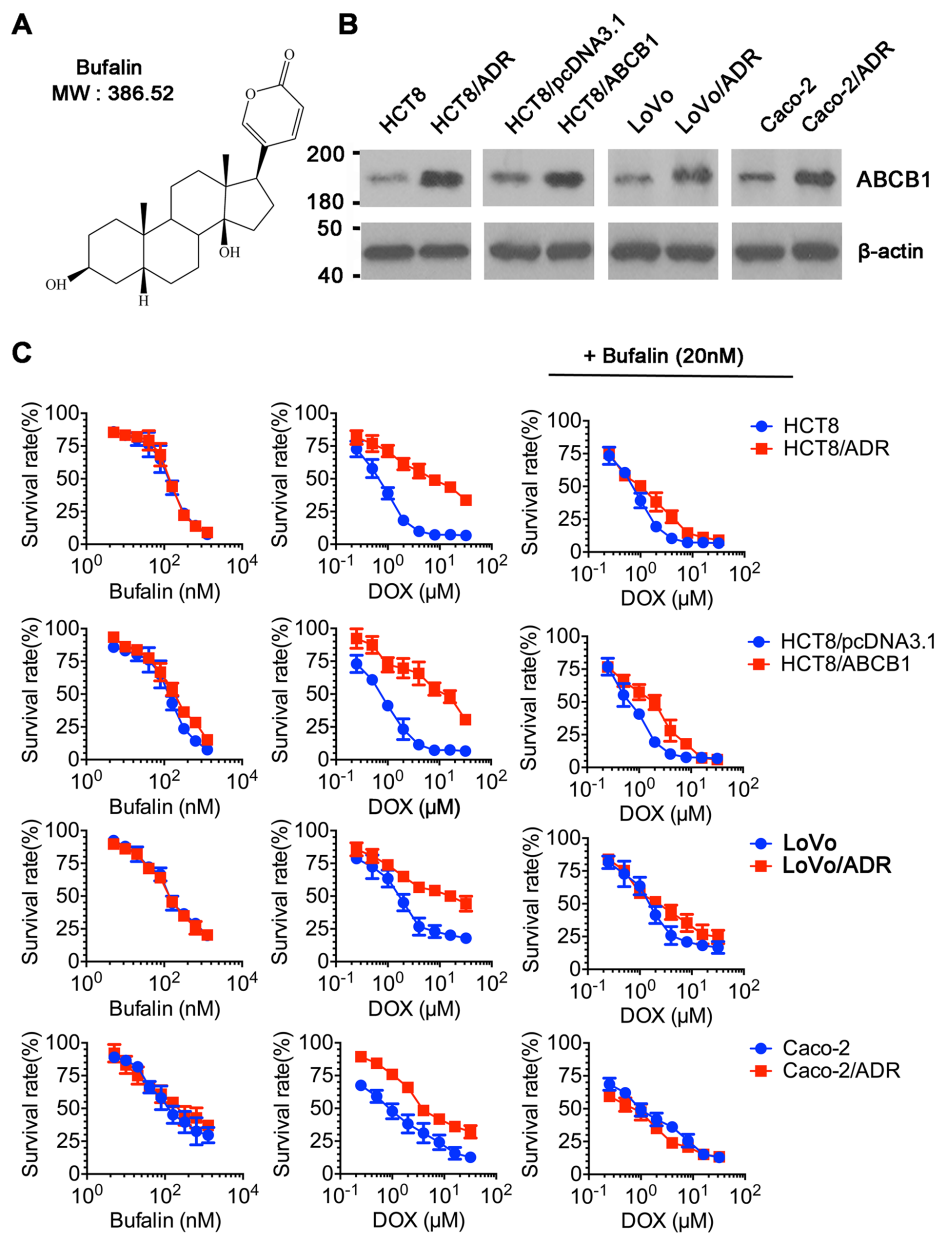
Cytotoxicity of BU in MDR and parental cell lines **(A)** Chemical structure of BU. **(B)** Western blot analysis of ABCB1 in drug-resistant cell lines and parental cells. β-actin was used as a loading control. **(C)** CCK-8 assay was used to evaluate cytotoxicity of BU in pairs of MDR and parental cell lines (left column); Cytotoxicity of DOX in pairs of MDR and parental cell lines (middle column); Cytotoxicity of DOX in the presence of BU in pairs of MDR and parental cell lines (right column). Representative curves were shown as cell survival rate verses concentration of compounds. Error bars represent the SD.

As shown in Figure 1, the protein levels of ABCB1 were overexpressed in LoVo/ADR, HCT8/ADR, HCT8/ABCB1 and Caco-2/ADR cells compared to their parental cells using western blot analysis. Subsequently, CCK-8 assays were conducted to obtain the non-toxic concentrations (survival rate higher than 80%) of BU in those cells. Results showed that BU concentrations up to20 nM were chosen as the maximum concentrations for the reversal assays. Next, further studies were examined whether BU could enhance the sensitivity of ABCB1-overexpressing cells to chemotherapeutic drugs. As shown in Table 1, the ABCB1-overexpressing Caco-2/ADR, LoVo/ADR, HCT8/ADR cells showed significant higher IC_50_ values to DOX (ABCB1 substrate) than their parental cells did. Treatment with BU at 5, 10, 20 nM lowered the IC_50_ ofABCB1-overexpressing cells towards DOX in a concentration-dependent pattern, while having no effect on the parental cells. The fold-reversal (RF) of BU to DOX was 2.98, 7.37 and 6.67 at 20 nM in Caco-2/ADR, LoVo/ADR, HCT8/ADR, respectively. To elucidate this specificity, we also examined the effect of BU in ABCB1-transfected HCT8/ABCB1 cells. In HCT8/ABCB1 and parental HCT8 cells, similar results were observed. In contrast, BU treatment at 20 nM did not affect the MDR to MIT (mitoxantrone, BCRP substance) or CDF (5(6)-carboxy-2′,7′-dichlorofluorescein, MRP2 substance) (Table 2). Taken together, our results indicated that BU could reverse ABCB1-mediated MDR in colorectal cancer cells.

**Table 1:** The cytotoxic of DOX on LoVo, LoVo/ADR, HCT8, HCT8/ABCB1, HCT8/pcDNA3.1 and HCT8/ABCB1 cells

Compound	IC_50_ (μM)	
	LoVo	LoVo/ADR
DOX	1.87 ± 0.15 (1.00)	16.28 ± 0.54 (1.00)
+BU (5.0 nM)	2.02 ± 0.19 (0.92)	4.54 ± 0.62(3.58)
+BU (10.0 nM)	1.65 ± 0.21 (1.13)	3.67 ± 0.22(4.43)**
+BU (20.0 nM)	1.58 ± 0.42 (1.18)	2.21 ± 0.30 (7.37)***
Verapamil (10μM)	1.76 ± 0.25 (1.06)	1.90 ± 0.28 (8.56)***
	**HCT8**	**HCT8/ADR**
DOX	0.55 ± 0.14 (1.00)	7.20 ± 0.80 (1.00)
+BU (5.0 nM)	0.56 ± 0.13 (0.98)	6.07 ± 0.35 (1.19)
+BU (10.0 nM)	0.54 ± 0.08 (1.02)	4.29 ± 0.36 (1.68)**
+BU (20.0 nM)	0.51 ± 0.10 (1.08)	1.08 ± 0.12 (6.67)***
Verapamil (10μM)	0.49 ± 0.05 (1.12)	0.81 ± 0.06 (8.89)***
	**HCT8/pcDNA3.1**	**HCT8/ABCB1**
DOX	0.67 ± 0.12(1.00)	13.35 ± 1.15 (1.00)
+BU (5.0 nM)	0.65 ± 0.08 (1.03)	10.33 ± 0.85 (1.29) **
+BU (10.0 nM)	0.68 ± 0.10 (0.98)	6.82 ± 0.64 (1.96) **
+BU (20.0 nM)	0.59 ± 0.11 (1.35)	2.13 ± 0.42 (6.27) ***
Verapamil (10 μM)	0.60 ± 0.07 (1.17)	1.22 ± 0.10 (10.94) ***

IC50 values are represented as mean ± SD of three independent experiments performed in triplicate. The fold-reversal of MDR (values given in parenthesis) was calculated by dividing the IC50 for cells with the anticancer drugs in the absence of bufalin or verapamil by that obtained in the presence of these two agents.

**p < 0.01 versus control group.

***p < 0.001 versus control group.

**Table 2:** Effect of Bufalin on the sensitivity of LoVo, LoVo/ADR, HCT8, HCT8/ADR, HCT8/ABCB1 cells to MIT/CDCF

Compound	IC50 (μM)	
	LoVo	LoVo/ADR
MIT(μM)	1.05 ± 0.09	1.23 ± 0.08
+ BU(20 nM)	1.10 ± 0.12	1.02 ± 0.10
CDCF(mM)	1.02 ± 0.11	1.13 ± 0.05
+ BU(20 nM)	0.98 ± 0.08	1.05 ± 0.12
	**HCT8**	**HCT8/ADR**
MIT(μM)	0.85 ± 0.15	1.02 ± 0.28
+ BU(20 nM)	0.82 ± 0.12	1.20 ± 0.19
CDCF(mM)	0.70 ± 0.10	0.96 ± 0.16
+ BU(20 nM)	0.72 ± 0.12	1.09 ± 0.20
	**HCT8/pcDNA3.1**	**HCT8/ABCB1**
MIT(μM)	0.88 ± 0.28	1.00 ± 0.20
+ BU(20 nM)	0.89 ± 0.12	0.95 ± 0.16
CDCF(mM)	0.91 ± 0.18	1.08 ± 0.14
+ BU(20 nM)	0.88 ± 0.09	1.12 ± 0.09

IC50 values are represented as mean ± SD of three independent experiments performed in triplicate.

### BU inhibited the efflux function of ABCB1

**Figure 2:**
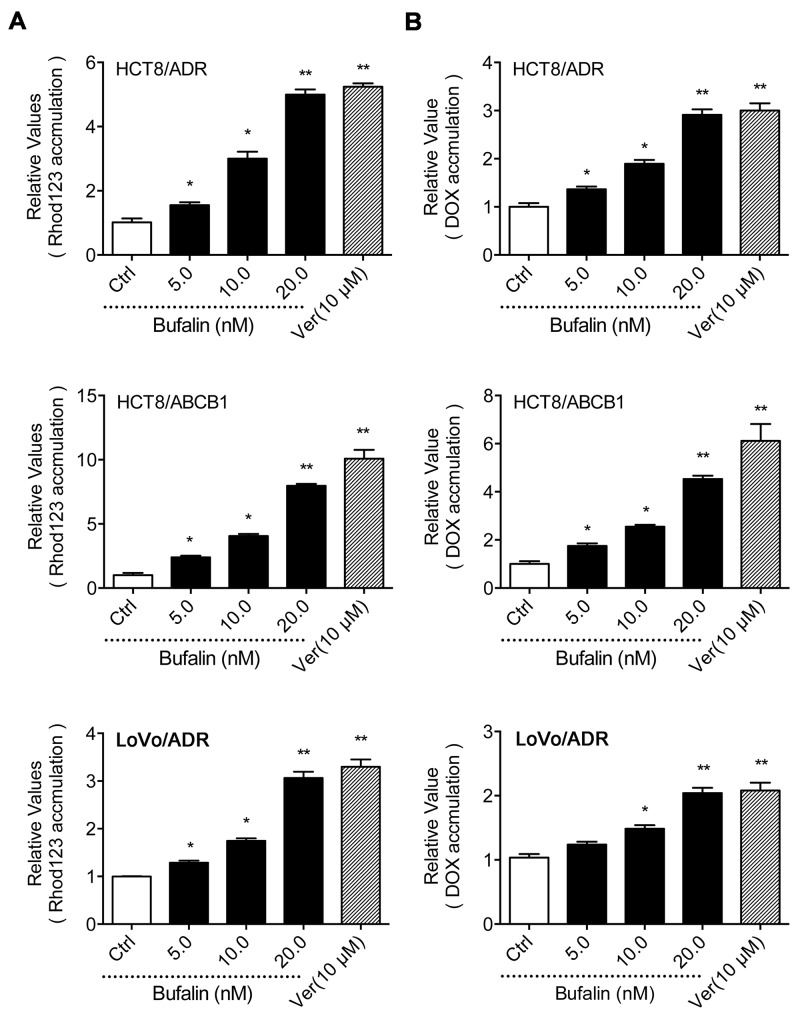
Effect of BU on the intracellular concentration of Rho 123 and DOX The accumulation of Rho 123 (A) and DOX (B) in the absence or presence of BU or verapamil (positive control) at 10 μM was measured by flow cytometry in HCT8/ADR, HCT8/ABCB1 and LoVo/ADR cells. The results are presented as fold change in fluorescence intensity relative to control MDR cells. Error bars represent the SD. Experiments were performed at least three independent times. *P< 0.05 versus control group, **P < 0.01 versus control group.

The accumulation of Rho 123(a substrate of ABCB1) and DOX assay was conducted to examine whether the reversal effect of BU was achieved by increasing the intracellular drug concentration. As shown in Figure 2, BU was able to increase the intracellular accumulation of Rho 123 and DOX in LoVo/ADR, HCT8/ADR and HCT8/ABCB1 cells in a dose-dependent manner. In detail, at the concentration of 5.0, 10.0 and 20.0 nM, BU increased the intracellular accumulation of Rho123 by 1.55-, 3.00-, 4.99-fold in HCT8/ADR cells, 2.39-, 4.06-, 7.97-fold in HCT8/ABCB1 cells and 1.29-, 1.75-, 3.07-fold in LoVo/ADR cells, respectively (Figure 2A). Similarly, BU at 5.0, 10.0 and 20.0 nM concentrations increased the intracellular accumulation of DOX by 1.37-, 1.90-, 2.91-fold in HCT8/ADR cells, 1.75-, 2.55-, 5.53-fold in HCT8/ABCB1 cells and 1,24-, 1.49-, 2.04-fold in LoVo/ADR cells, respectively (Figure 2B). These findings suggested that BU could circumvent MDR by inhibiting the efflux function of ABCB1.

### BU inhibited ABCB1-mediated drug transport

Permeability coefficients (Papp) tested in Caco-2 cell monolayer models were shown in Table 3. The absorbable permeability coefficient (Papp (A to B)) of DOX obtained from the apical (A) to the basoleteral(B) side and the secretory permeability coefficient (Papp (B to A)) determined from the basoleteral(B) to the apical (A) side were measured. After treatment with BU (20 nM), the Papp (A to B) value was increased from 0.68±0.18 to 1.02±0.20×10^−6^ cm/s and the Papp (B to A) value was decreased from 3.91±0.68 to 3.35±0.34×10^−6^ cm/s. Efflux ratio (ER) values were used to evaluate the potential ability of active efflux transport. Compared with the result in the presence of BU, the ER value of DOX was reduced (5.75 vs 3.28, *P* < 0.01), suggesting that BU could reverse ABCB1-mediated drug transport.

**Table 3:** Papp and ER values of DOX in the absence or presence of Bufalin

Compound	Papp (×10^−6^)		ER
	A to B	B to A	
DOX	0.68 ± 0.18	3.91 ± 0.68	5.75
DOX + Bufalin (20 nM)	1.02 ± 0.20**	3.35 ± 0.34*	3.28**
DOX + Verapamil (10 μM)	1.23 ± 0.05**	2.81 ± 0.16**	2.28***

Data are presented as mean values ± SD (n = 3).

** P < 0.01, comparing with DOX group.

*** P < 0.001, comparing with DOX group.

### BU enhanced the doxorubicin-effect by inhibiting ABCB1 *in vivo*

**Figure 3:**
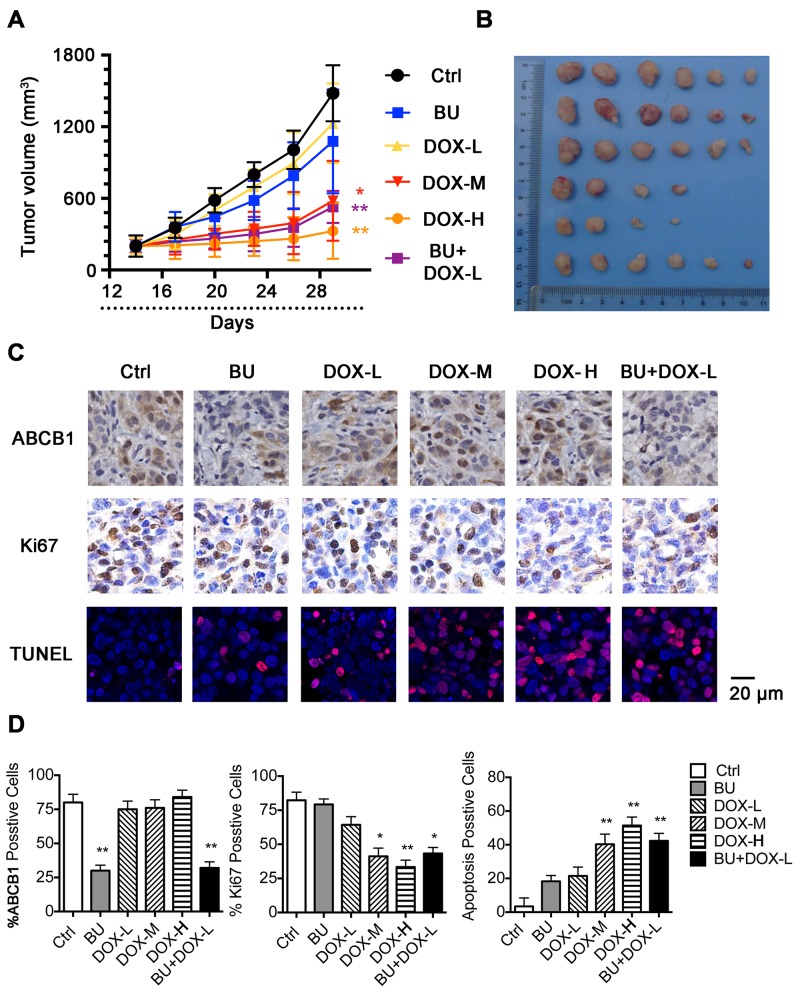
Potentiation of the antitumor effects of DOX by BU in a nude mice xenograft model **(A)** Changes in tumor volume with time after tumor cell inoculation. Points, mean tumor volume for each group of six mice after implantation; bars, SD. **(B)** Tumor size. The photograph was taken on the 29th day after implantation. The various treatments were as follows: control; BU(0.1 mg/kg, i.p., q3d×5); DOX (0.1 mg/kg, i.p., q3d×5); DOX (0.5 mg/kg, i.p., q3d×5); DOX (1.0 mg/kg, i.p., q3d×5) and DOX (0.1 mg/kg, i.p., q3d×5) plus BU (0.1 mg/kg, i.p., q3d×5, given 1hr before DOX administration). **(C)** IHC for ABCB1, Ki67 and Immunofluorescence for TUNEL assay were performed in tumor tissues at the end of experiments. Scale bar represents 20 μm. **(D)** The positive rate of ABCB1, Ki67 and TUNEL are based on IHC. *P< 0.05, comparing with control group,**P< 0.01, comparing with control group.

In order to investigate the efficacy of BU to reverse MDR *in vivo*, we established HCT8/ADR cells nude mouse xenograft model to test effects of DOX (0.1, 0.5, 1.0 mg/kg), BU 0.1 mg/kg, and the combination of BU (0.1mg/kg) and DOX (0.1mg/kg) on the tumor growth inhibition. As shown in Figure 3A&3B, DOX was able to inhibit tumor growth with a concentration-dependent pattern. No significant difference existed in tumor size between groups treated with saline, BU 0.1 mg/kg or DOX 0.1 mg/kg alone. Thus, we chose DOX 0.1 mg/kg as the safe concentration to combine with BU (0.1 mg/kg). However, tumor size in the combination group of BU (0.1 mg/kg) and DOX (0.1 mg/kg) was smaller than groups treated with them alone. As shown in Figure 3C&3D, the results showed that BU could inhibit ABCB1 expression *in vivo*, and the cell proliferation of the combined group was decreased compare with groups treated with them alone (Ki67 level), the apoptosis rate was increased (TUNEL assay). These data demonstrated that BU could enhance the antitumor effect of chemotherapy agents by decreasing the ABCB1 expression *in vivo*.

**Figure 4:**
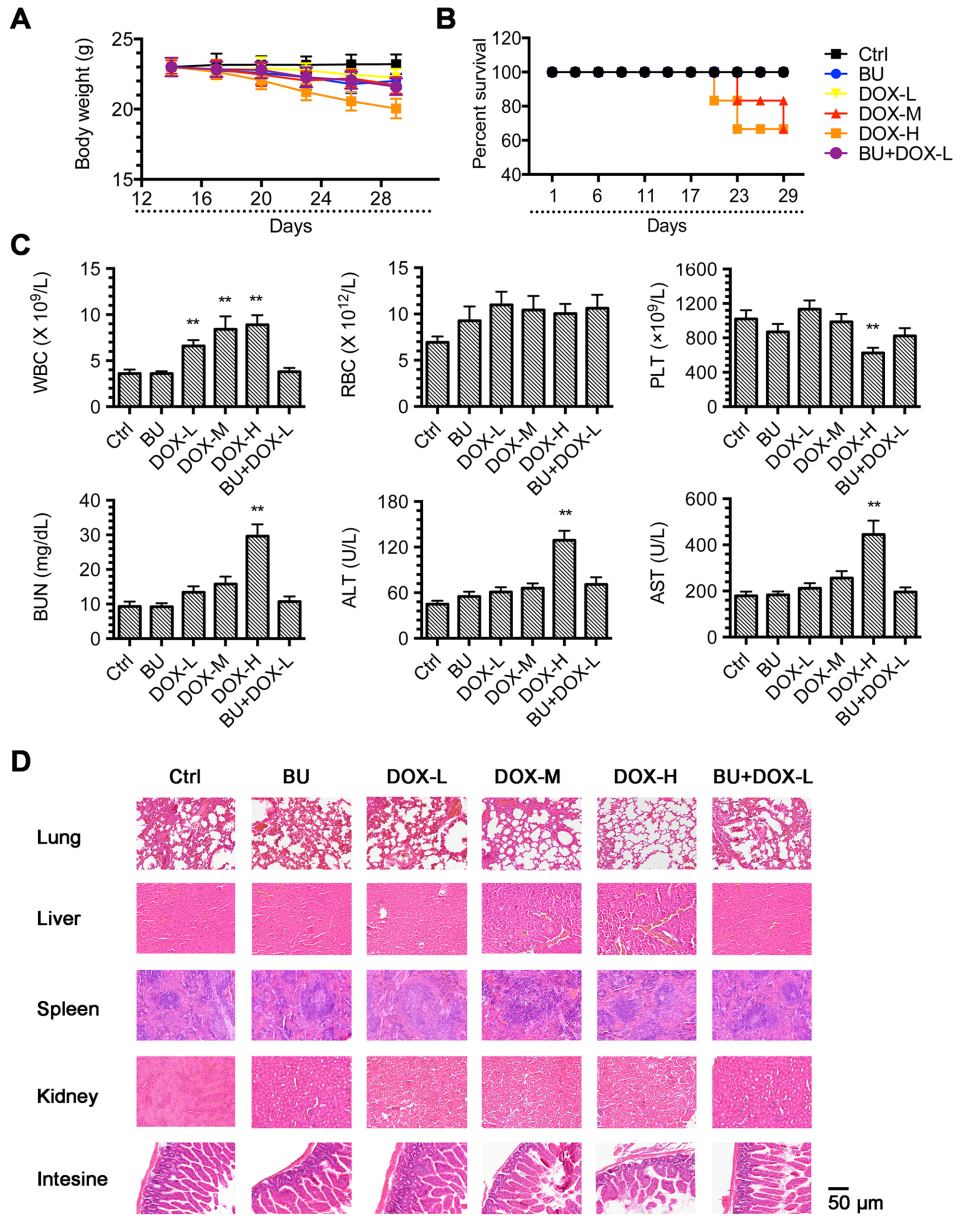
Toxicity analysis **(A)** Changes in mice weight with time after tumor cell inoculation. Points, mean mice weight for each group after implantation; bars, SD. **(B)** The survival curve of mice treated with each group. **(C)** Blood analysis of mice after treatment with BU 0.1mg/kg, DOX 0.1mg/kg, DOX 0.5mg/kg, DOX 1.0mg/kg or DOX 0.1mg/kg +BU 0.1mg/kg. Error bars represent ± S.D. **(D)** H&E histology of various organs after treatment of Nu/Nu mice bearing HCT8/ADR cancer xenografts with DOX, BU or in combination(n= 6 mice per group). Scale bar represents 50 μm.

Meanwhile, significant toxicity (2 deaths out of 6 mice) was observed in the DOX 0.5 mg/kg and DOX 1.0 mg/kg groups by weight loss (Figure 4A&4B). Both alanine transaminase (ALT) and aspartate transaminase (AST) were significantly elevated showed high concentration DOX cause the hematologic disorders, and also pathological analysis indicated the organ function abnormalities like microsteatosis in the liver and atrophic white pulp in the spleen. But in the BU and DOX combination group, which had the same anti-tumor effect, did not cause weight loss or other toxicities (Figure 4C&4D). These data demonstrated that BU could reduce the doxorubicin-toxicity by increasing the doxorubicin-effect *in vivo*.

### BU stimulated ATPase activity of ABCB1

**Figure 5:**
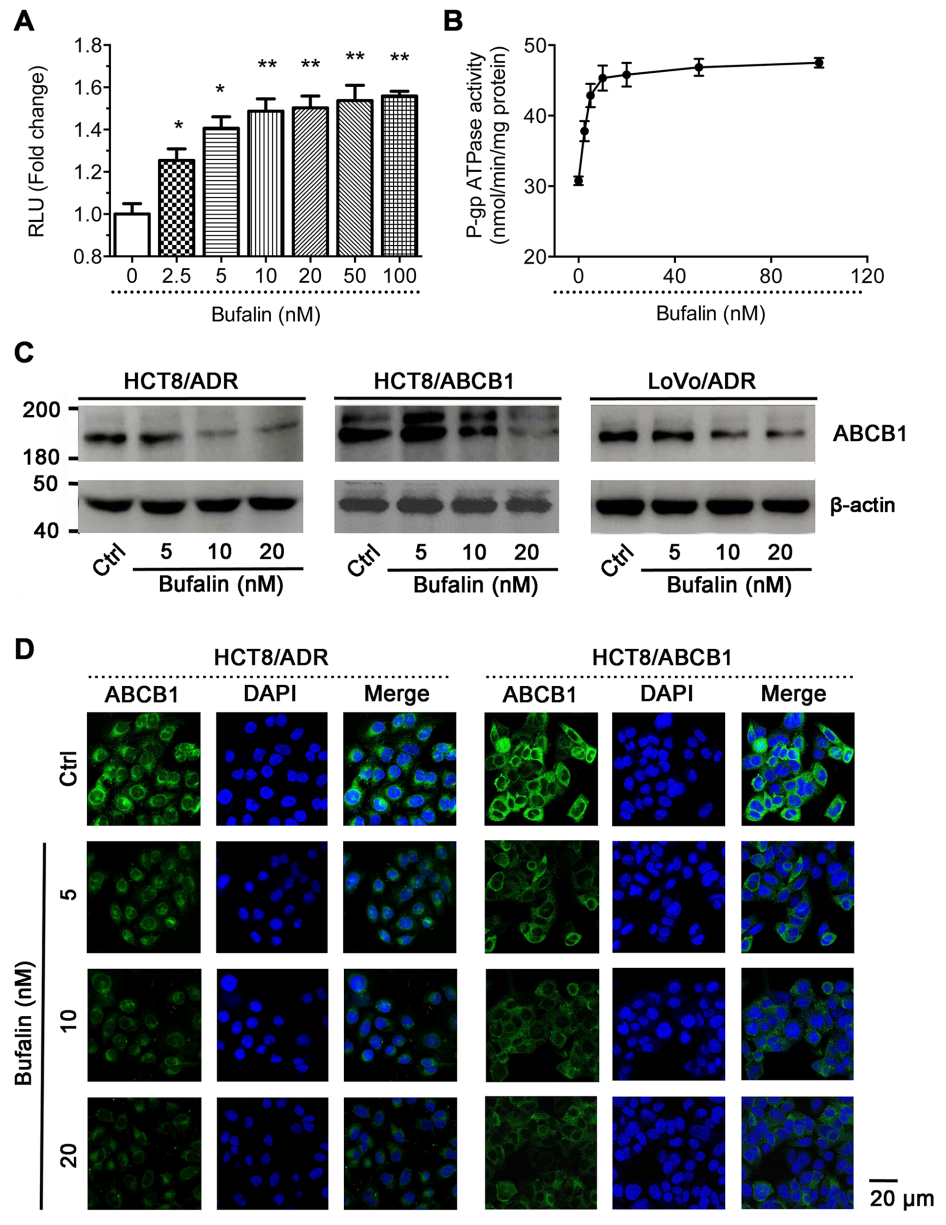
Effect of BU on ABCB1 ATPase activity and the protein expression of ABCB1 **(A)** Effect of BU on RLU values in HCT8/ADR. **(B)** Effect of BU on verapamil stimulated ATPase activity in HCT8/ADR. **(C)** Western blot analysis of ABCB1. Effect of BU on the protein expression of ABCB1 in MDR cells. **(D)** Subcellular localization of ABCB1 in ABCB1-overexpressed HCT8/ADR and HCT8/ABCB1 cells. Effect of BU (5, 10, 20 nM) on the localization of ABCB1. Scale bar, 20 μm, DAPI (blue) counterstains the nuclei.

The drug efflux function of ABCB1 is dependent on the energy, which released from ATP hydrolysis. Thus, we measured effect of BU on ABCB1-mediated ATP hydrolysis in HCT8/ADR cell line. As shown in Figure 5A&5B, BU increased Ver-stimulated ATPase activity dose-dependently. This ATPase data indicated that BU may be a substrate for ABCB1.

### BU inhibited the expression of ABCB1 in ABCB1-overexpressing cells

In order to investigate whether BU can change the expression of ABCB1 *in vitro*, we determined the effect of BU on ABCB1 protein in ABCB1-overexpressing cells with western blot assay. As shown in Figure 5C, treatment with BU at 5, 10, 20 nM significantly deceased the expression level of ABCB1 dose-dependently in LoVo/ADR, HCT8/ADR, HCT8/ABCB1 cells. The data showed consistent trend by IHC staining in HCT8/ADR xenografts (Figure 3C&3D).

To demonstrate whether BU can influence the subcellular localization of ABCB1 transporters, we performed an immunofluorescence staining assay in ABCB1-overexpressing cells. As shown in Figure 5D, the location of ABCB1 in the HCT8/ADR and HCT8/ABCB1 cells which treated with BU (5, 10, 20 nM) did not significantly change when compared to the control, but the expression of ABCB1 was decrease. These results indicated that the reversal effect of BU on ABCB1-mediated MDR was probably due to the inhibition of ABCB1 expression.

### Binding model between BU and ABCB1

**Figure 6:**
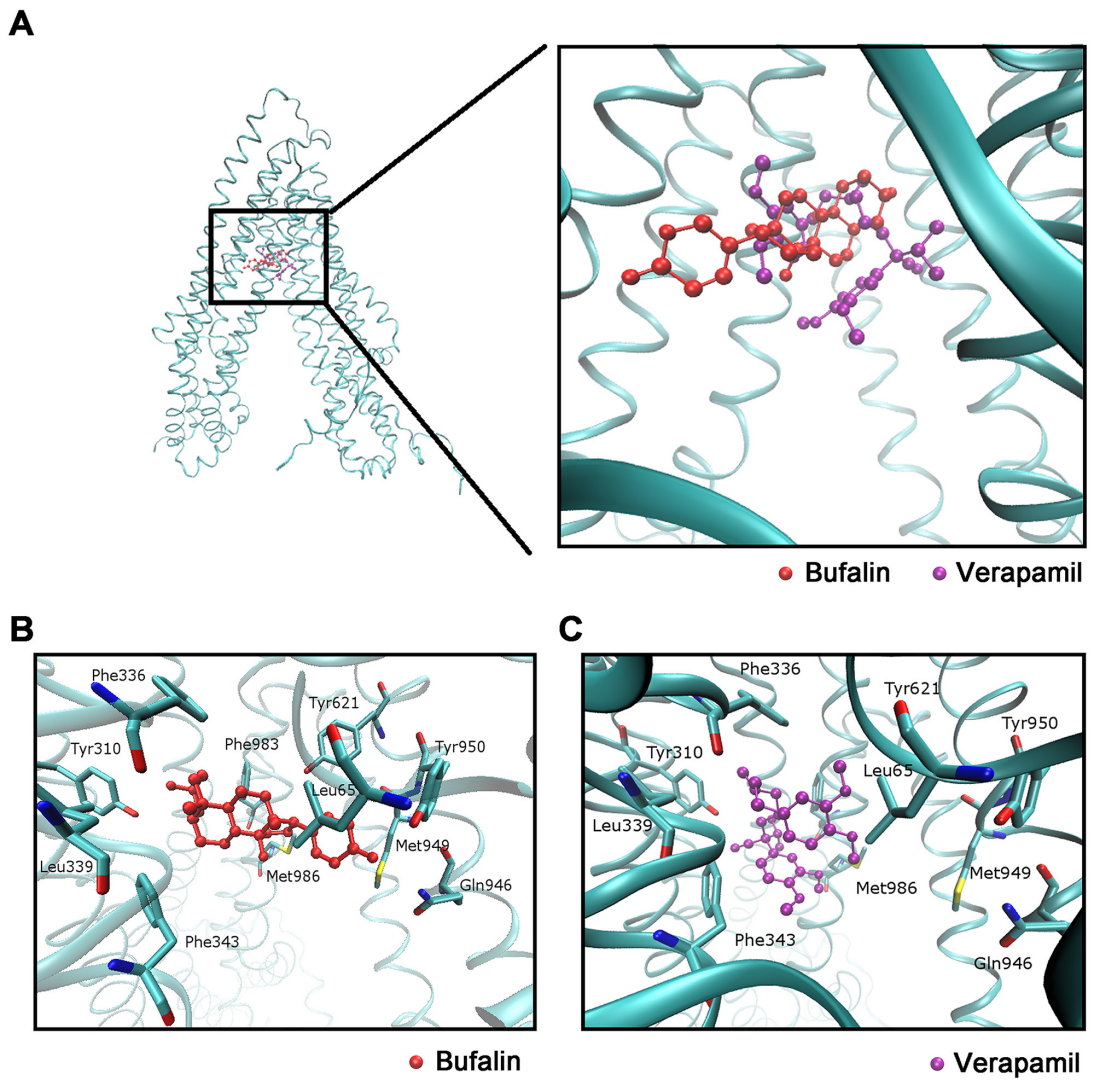
Molecular modeling of binding of BU or Verapamil to homology ABCB1 **(A)** Location of BU (red) and Verapamil (purple) molecules in the ABCB1 internal cavity. **(B)** The docked conformation of BU within the binding cavity of ABCB1 is shown as a ball and stick model. Important residues are depicted as sticks with the atoms colored as follows: carbon, green; hydrogen, white; nitrogen, blue; oxygen, red; sulfur, yellow; whereas BU is shown with the same color scheme as above except the carbon atoms are presented in red. **(C)** The docked conformation of Verapamil. Color scheme is same as panel **(B)** except carbon atoms of Verapamil are presented in purple.

The existing results have indicated that BU may have direct interactions with ABCB1 transporter. To confirm our hypothesis, we performed a molecular docking simulation to determine the binding model between BU and human ABCB1, the 3D structure of human ABCB1 was generated by homology modeling, and BU was successfully docked into ATP binding domain of this model which is as same pocket as the ABCB1 inhibitor Verapamil binding (Figure 6A). Furthermore, the FullFitness scores of the best docked poses were -2987.85 kcal/mol for BU and -2988.37 kcal/mol for Verapamil, which translates to estimated free energies of binding of -7.85 kcal/mol for BU and -8.35 kcal/mol for Verapamil, showed that BU has the same binding capacity as Verapamil. The docked pose of BU and Verapamil is shown in Figure 6B&6C, and they were stabilized by hydrophobic residues Leu65, Tyr310, Phe336, Leu339, Phe343, Tyr621, Glu946, Met949, Tyr950, and Met986.

## DISCUSSION

Colon cancer is a commonly observed malignant tumour in clinical settings. It ranks third in cancer-related deaths, shows a trend of yearly increase in incidence, and has an early age of onset [23]. The ineffectiveness and lethality of cancer chemotherapy can be attributed to the intrinsic resistance of tumours or their propensity to develop resistance during chemotherapy. [24] Therefore, developing effective drugs for reversing MDR in colon cancer has important research and clinical significance.

BU, which is extracted from Chinese toad venom, is a toxic ligand with maximal antitumour effects and is a bufadienolide[25–27]. Modern research has found BU to have strong anti-tumour effects. Its mechanism involves the inhibition of tumour cell growth and the induction of apoptosis of tumour cells[28, 29]. This paper reports, for the first time, an investigation of the function and mechanism of BU in reversing ABCB1-mediated MDR.

Firstly, we used drug-resistant colon cancer cells that overexpressed ABCB1, LoVo/ADR, and HCT8/ADR as models. The CCK8 assay was used to analyse the reversal of drug-resistant cell lines by BU. The results indicated that BU at safe concentration significantly increased the sensitivity of drug-resistant cells to DOX (the substrate of ABCB1), but had no effect on MRP and BCRP substrate drug (Figure 1) [30, 31]. These results indicated that the reverse efficacy of BU on MDR was related to its interaction with ABCB1. To further elucidate their specificity, we established ABCB1 transfected HCT8/ABCB1 cell lines. Similarly, the results showed the same trend as those in drug-selected MDR cells. These data together demonstrate that BU can specifically inhibit ABCB1-mediated MDR in both drug-selected LoVo/ADR, HCT8/ADR cells and transfected HCT8/ABCB1 cells.

In order to investigate the mechanism of BU's reversal effect, we examined the intracellular accumulation of Rho123 and DOX in ABCB1-overexpressing cells by flow cytometry (Figure 2). Our results indicated that BU could increase the intracellular level of Rho123 and DOX in a concentration-dependent manner. Furthermore, the Caco-2 monolayer permeability assay demonstrated that the ability of BU to promote the monolayer absorption of DOX and inhibit the efflux of DOX. These results show that BU can increase the accumulation of drugs in resistant cells and decrease drug efflux by inhibiting ABCB1-mediated drug transport and, thereby, achieve the goal of overcoming MDR.

More meaningfully, HCT8/ADR cells xenograft model in nude mice further proved the efficacy of BU *in vivo*. The safety of MDR reversal agents is an important factor which influences their further research and development. The toxicity analysis indicated that BU at safe dose (0.1mg/kg) did not induce any toxicity on mice. Taken together, these results suggest that BU may have the potential to be used as a safe and effective MDR reversal agent (Figures 3&4).

As reported, the reversal of ABCB1-mediated MDR could be achieved either by down-regulation the expression of ABCB1 and/or inhibiting its transport function (Figure 5) [32–34]. Western blot analysis showed that BU could attenuate the protein expression of ABCB1 in MDR cells. The same results were obtained in ABCB1-transfected HCT8/ABCB1 cells. Herein, we found that BU at safe dose could effectively reverse MDR by decreasing the expression of ABCB1.

Furthermore, we subsequently performed ATPase activity assay to examine whether the reversal effect of BU was associate with inhibition of the transport function of ABCB1. ABC transporters are consisting of two transmembrane domains (TMDs) and two nucleotide-binding domains (NBDs)[35]. The ABCB1 transporter substrate depends on the energy released by the hydrolysis of ATP to adenosine diphosphate (ADP) and phosphate (Pi) [36]. The ABCB1 transporter substrate undergoes the following process: First, the substrate enters the internal drug-binding pocket and causes a conformational change in the transmembrane domain (TMD). Next, the drug is transported out of the membrane, which leads to automatic dissociation of ADP. Additional ATP hydrolysis occurs, and after the ADP dissociation, ABCB1 returns to its original state[37]. ABCB1 regulators that bind to the TMD are known as competitive inhibitors, while those that block NBD are known as non-competitive inhibitors[38]. The results of an ATPase assay demonstrated BU's ability to stimulate ATPase activity, indicating that it is a competitive inhibitor capable of binding to the drug-binding sites of ABCB1. Docking experiments also showed that BU binds efficiently to the drug-binding pocket. This result supports the hypothesis that BU could inhibit the transport function of ABCB1 by competitively binding to the substance binding domain (Figure 6).

Wei Gu et al. reported that BU could reversed MDR in human hepatocellular carcinoma cells through multiple pathways, including inhibit drug efflux function via down-regulation of MRP1, induce apoptosis and arrested the cell cycle at the G0/G1 phase. However, their results were hardly related to alter the protein expressing of ABCB1[20]. By comparison, we used colorectal cancer cell model, rather than hepatocellular carcinoma cell model, and the different results between the two studies may be result from differences of cells model.

In the preliminary experiment, we chosed six kinds of active ingredients of Chansu, including bufalin, cinobufagin, resibufogenin, bufotalin, arenobufagin and gamabufotalin to detect their effects on MDR. Our results indicated that bufalin and cinobufagin could effectively reverse MDR in colon cancer with different mechanisms. Interesting, more studies should be taken about structure-activity relationship of venenum Bufonis monomers.

In conclusion, this study for the first time demonstrated that BU reverse ABCB1-mediated MDR by inhibiting transport function of ABCB1 and down-regulate its protein expression. Our results indicated that BU as combination therapy may be a useful strategy to overcome clinical resistance in colorectal cancer chemotherapy.

## MATERIALS AND METHODS

### Materials

Bufalin, Doxorubicin (DOX), Rho-123, verapamil and Lucifer yellow were obtained from Sigma–Aldrich Chemical Co. (St. Louis, MO, USA). Minimum Essential Media (MEM), fetal bovine serum (FBS), non-essential amino acids (NEAA), Ham's F-12K (Kaighn’s) Medium (F12K), and Hank's balanced salt solution (HBSS), were obtained from Gibco BRL (Carlsbad, CA, USA). RPMI 1640 and phosphate-buffered saline (PBS) were from Hyclone (Thermo Scientific, Logan, UT). Annexin V-FITC Apoptosis Detection Kit was purchased from BD Biosciences (Beijing, China). Cell Counting Kit-8 (CCK-8) was purchased from Dojindo (Kumamoto, Japan). A ABCB1 ATPase assay system was purchased from Promega (Madison, WI, USA). The primary antibodies for ABCB1 (#13342) and β-actin (#3700) were sourced from Cell Signaling Technology (Boston, USA). The secondary antibodies were obtained from Santa Cruz Biotechnology (Santa Cruz, CA, USA).

### Cell lines and culture conditions

The human colon cancer cell lines LoVo, HCT8, Caco-2 were obtained from the Cell Bank of the Chinese Academy of Sciences. DOX-selected ABCB1-overexpression LoVo/ADR, HCT8/ADR, Caco-2/ADR cell lines were were purchased from Shanghai Yan Sheng Industrial Co., LTD. All cell lines were used for reversal study and were cultured in RPMI-1640, MEM or F12K containing 10% FBS at 37°C in a humidified atmosphere of 5% CO_2_. All the DOX-selected ABCB1-overexpression cells were seeded in a medium containing 1μM of DOX to maintain their drug resistance phenotype.

HCT8/pcDNA3.1 and HCT8/ABCB1 cells were established by transfecting HCT8 with empty pcDNA3.1 or vector containing the full length ABCB1, and were cultured in medium with 2 mg/ml G418.

### Cell cytotoxicity by CCK-8 assay

The CCK-8 assay was used to detect the sensitivity of the cells to anticancer drugs. Cells (1×10^4^/well) were seeded into (100 μl) 96-well plates and cultured overnight at 37°C. Different concentrations of anticancer drugs in the absence or presence of inhibitors were added into the designed wells. After 48h of incubation, 10 μl of CCK-8 solution was added to each well, cells were further cultured for 1-4 h. The absorbance was determined at 450 nm by Thermo Varioskan Flash (Thermo Scientific, MA, USA). The IC_50_ values were estimated by GraphPad Prism 5.0 (La Jolla, CA, USA). Verapamil (10 μM) was used as a positive control.

### DOX and Rho123 accumulation assay by flow cytometry

The effect of BU on accumulation of DOX and Rho123 in LoVo/ADR, HCT8/ADR, HCT8/ABCB1 cells were measured by flow cytometry. After treating with BU for 48h, cells were cultured with DOX (5 μg/ml) or Rho 123(1 μg/ml) for another 90 min, respectively. Finally, samples were collected and washed twice with PBS, and analyzed by flow cytometry (BD Biosciences, San Jose, CA). Verapamil (10 μM) was used as a positive control.

### ABCB1-mediated drug transport assay

Caco-2 cells were seeded on a Costar 12-well Transwell Permeable Supports plates (0.4 μm pore size, 1.13 cm^2^ of growth area, 12 mm diameter) at a density of 5.0×10^4^cells/cm^2^. Replaced the medium every other day and incubate plates at 37°C for 21 days. Transepithelial electrical resistance (TEER) and Lucifer yellow permeability (LY%) was measured to evaluate the integrity of the monolayer. TEER and LY% values above 250 Ω·cm^2^ and below 1% were considered as acceptable, respectively. Cells were washed 3 times with HBSS buffer and preincubated for 15 min. Test drugs were diluted in HBSS with 0.5 ml added to the apical side (A) or 1.5 ml to the basolateral side (B). At 0, 15, 30, 45, 60, 90 and 120 min, 0.1 ml samples were withdrawal from the opposite sides and supplied with fresh medium. Drug transport studies were conducted at 20 μM DOX with or without BU(20 nM) in HBSS buffer. Verapamil (10 μM) was used as a positive control. Analyte concentrations were determined by Thermo Varioskan Flash.

The apparent permeability coefficient (Papp) was determined for both A to B and B to A directions by the following formula: $\text{Papp}=\frac{dC}{dt}\times \frac{V}{AC_{0}}$ in which A is the area of filter membrane, C_0_ represents the initial concentration of drug, dC/dt is the change of concentration of drug in the period of incubation time, and V is the volume of the receiver chamber. The efflux ratio (ER) was calculated from: $\text{ER}=\frac{P_{app}(B\;to\;A)}{P_{app}\;(A\;to\;B)}$

### ABCB1 ATPase assay

The ATPase activity of ABCB1 of HCT8/ADR cell was carried out using Pgp-Glo™ assay system following the instructions of the manufacturer. Briefly, the recombinant human ABCB1 membranes were incubated with or without vanadate, in different concentrations of BU (2.5, 5, 10, 20, 50, 100 nM) at 37°C for 5 min. Then MgATP (25 mM) was added to each well and incubated at 37°C for 40 min. Subsequently, luminescence was initiated with ATP detection reagent. After incubation at room temperature for 20 min, to allow the luminescent signal to develop, the untreated, white, opaque, 96-well plate (Corning, Iowell, MA) was read on Thermo Varioskan Flash.

### Western blot analysis

Cell lysates were prepared in RIPA buffer in the presence of 1% proteinase inhibitor. Proteins (40 μg) were separated on 8%-15% SDS-PAGE and transferred to PVDF membranes. After blocking using 5 % BSA, membranes were incubated with primary monoclonal antibodies: ABCB1 (1:1000) and β-actin (1:2500) overnight at 4 °C and then incubated with HRP-conjugated secondary antibodies (1:5000) for 1 h. Bands were detected using enhanced chemiluminescence detection (GE Healthcare Lifesciences, Pittsburgh, PA, USA).

### Immunofluorescence for ABCB1 by confocal microscopy

HCT8/ADR and HCT8/ABCB1 cells were seeded on cover glasses and cultured with different concentrations of BU (5, 10, and 20 nM) for 48 h. Then cells were fixed with 4% (v/v) paraformaldehyde for 10 min at 37°C. Subsequently, cells were covered with BSA (2 mg/ml) for 1 h followed by incubation with primary antibody against ABCB1 (1:800) at 37°C for 2 h. After washing three times with PBS, cells were further incubated with FITC-conjugated anti-mouse IgG for 1 h. Finally, nuclei were stained with DAPI for 3 min. Fluorescent signals were detected by confocal fluorescence microscopy (LEICA DM IRB; Leica, Wetzlar, Germany).

### Nude Mouse Xenograft Model

Athymic nude mice (BALB/c-nu/nu) of 6-8 weeks were used to establish HCT8/ADR xenograft model following the approval of the Administrative Panel on Laboratory Animal Care of the Putuo District Center Hospital. Briefly, HCT8/ADR (1×10^7^) cells were resuspended in 200 μl PBS and implanted s.c. under the shoulder in the nude mice. When the tumor volumes reached 150-200 mm^3^, the mice were randomized into six groups (6 in each group): control; BU(0.1 mg/kg, i.p., q3d×5); DOX (0.1 mg/kg, i.p., q3d×5); DOX (0.5 mg/kg, i.p., q3d×5); DOX (1.0 mg/kg, i.p., q3d×5) and DOX (0.1 mg/kg, i.p., q3d×5) plus BU (0.1 mg/kg, i.p., q3d×5, given 1hr before DOX administration). The mice were weighed and their tumor sizes were measured every 3 days. Tumor volumes (V) were calculated using the formula: V = 1/2 × larger diameter × (smaller diameter)^2^. When tumor weights grew to 1 g, the mice were euthanized and whole blood, tumor and normal tissues were harvested for further examination. Tumor tissues were fixed in formalin, embedded in paraffin and analyzed by Immunohistochemical (IHC) staining for TUNEL, Ki67 and ABCB1 in Molecular Pathology Lab.

### Toxicity analysis

Normal tissues (heart, liver, lung, spleen, kidney, intestine) were harvested for H&E histology studies. Venous blood samples were collected in EDTA-coated tubes for hematology studies. Samples were analyzed for white blood cells (WBC), red blood cells (RBC), platelets (PLT), aspartate aminotransferase (AST), alanine aminotransferase (ALT) and blood urea nitrogen (BUN) in the clinical laboratory at our hospital.

### Molecular modeling

The X-ray crystal structure of mouse ABCB1 (PDB ID: 4M2S), obtained from the RCSB Protein Data Bank, was used as the template to build the homology model of human ABCB1 [39]. BU was docked to the homology model using SwissDock as described [40]. The residues around the ligand are shown in the figure, which was created using VMD.

### Statistics

Statistical analysis was performed using GraphPad Prism 5.0 software. Differences between groups were analyzed by two tailed Student's t-test. *P* values below 0.05 were considered significant.

## Abbreviations

MDR: multidrug resistance
ABC: ATP-binding cassette
ABCB1: ABC transporter-subfamily B member 1
ABCC1: ABC transporter-subfamily C member 1
ABCG2: ABC transporter-subfamily G member 2
P-gp: (ABCB1) P-glycoprotein
BU: bufalin
DOX: doxorubicin
Rho 123: Rhodamine 123
TMD: transmembrane domain
NBD: nucleotide-binding domain
ADP: adenosine diphosphate
Pi: phosphate
IHC: immunohistochemical.
